# Factors contributing to severe scoliosis after open chest surgery for congenital heart disease: a case–control analysis

**DOI:** 10.1007/s43390-024-01009-4

**Published:** 2024-11-12

**Authors:** Ichiro Kawamura, Toru Yamaguchi, Haruhisa Yanagida, Hiroyuki Tominaga, Takuya Yamamoto, Kentaro Ueno, Noboru Taniguchi

**Affiliations:** 1 Department of Orthopaedic Surgery, Graduate School of Medicine and Dental Science, Kagoshima University, 8-35-1 Sakuragaoka, Kagoshima, 890-8520 Japan; 2 Department of Orthopaedic and Spine Surgery, Fukuoka Children’s Hospital, Fukuoka, Japan; 3 Department of Orthopaedic Surgery, Kagoshima City Hospital, Kagoshima, Japan; 4 Department of Pediatrics, Graduate School of Medicine and Dental Science, Kagoshima University, Kagoshima, Japan

**Keywords:** Congenital heart disease, Open chest surgery, Scoliosis, Children

## Abstract

**Purpose:**

Previous reports have identified factors associated with open chest surgery for congenital heart disease (CHD) and scoliosis. However, these reports included conditions such as Down syndrome and Marfan syndrome, which involve both cardiac disease and scoliosis. The relationships between these factors and open chest surgery remain unclear. This study aimed to identify factors contributing to severe scoliosis in CHD patients who have undergone open chest surgery.

**Methods:**

Seventy-four post-CHD surgery patients with severe scoliosis (Scoliosis group) and 30 post-CHD surgery patients without scoliosis (NS group), excluding those with any syndrome or intellectual disability, were retrospectively analyzed. Patient background characteristics and radiographic parameters were compared between the NS and Scoliosis groups. Furthermore, the patients in the Scoliosis group were classified into three categories, namely, mild scoliosis, moderate scoliosis, and severe scoliosis, and the results were compared among the four groups.

**Results:**

Eighteen patients in the NS group and 63 in the Scoliosis group met the inclusion criteria. Compared with the NS group, the Scoliosis group included significantly more girls and patients who had younger ages at first CHD surgery and multiple open chest surgeries. Severe scoliosis progression was observed in patients who underwent multiple surgeries for severe CHD with cardiomegaly.

**Conclusions:**

Progression to severe scoliosis was noted in patients with younger ages at first CHD surgery and those who underwent multiple surgeries for severe CHD. Assessing spinal deformities should be a key aspect of postoperative care for CHD, particularly in patients with severe CHD who are undergoing multiple chest surgeries.

**Level of evidence:**

III.

## Introduction

The prevalence of idiopathic scoliosis alone is 2–3% of the general population [1, 2], whereas the prevalence of scoliosis with congenital heart disease (CHD) is reported to be 10–42%, and 2.5–3% of these patients will develop severe scoliosis [3–5]. Several risk factors for scoliosis after CHD surgery, such as sex, age at CHD surgery, and open chest procedures, have been discussed [6–11], but the causes and factors associated with progression are still unclear. There have also been reports of female sex, syndromes, and intellectual disability as factors in the progression to severe scoliosis [5]. However, there is concern about the possibility of syndromes accompanied by both cardiac disease and scoliosis, such as Down syndrome and Marfan syndrome. This may be confounded by the presence of a syndrome or by the involvement of open chest surgery for CHD. The prevalence of intellectual disability also cannot rule out the possibility of an undiagnosed genetic syndrome or chromosomal abnormality. Thus, the influence of open chest surgery for CHD on scoliosis progression is even more uncertain. The purpose of the present study was to identify the factors that contribute to the development of severe scoliosis in individuals after undergoing open chest surgery for cardiothoracic abnormalities after the exclusion of patients with obvious syndromes and intellectual disability.

## Materials and methods

The institutional review board of the author’s affiliated institution approved this retrospective observational study protocol, which employed an opt-out policy. A retrospective review of radiographic and clinical data was performed in accordance with the ethical standards of the 1964 Declaration of Helsinki.

**Fig. 1:**
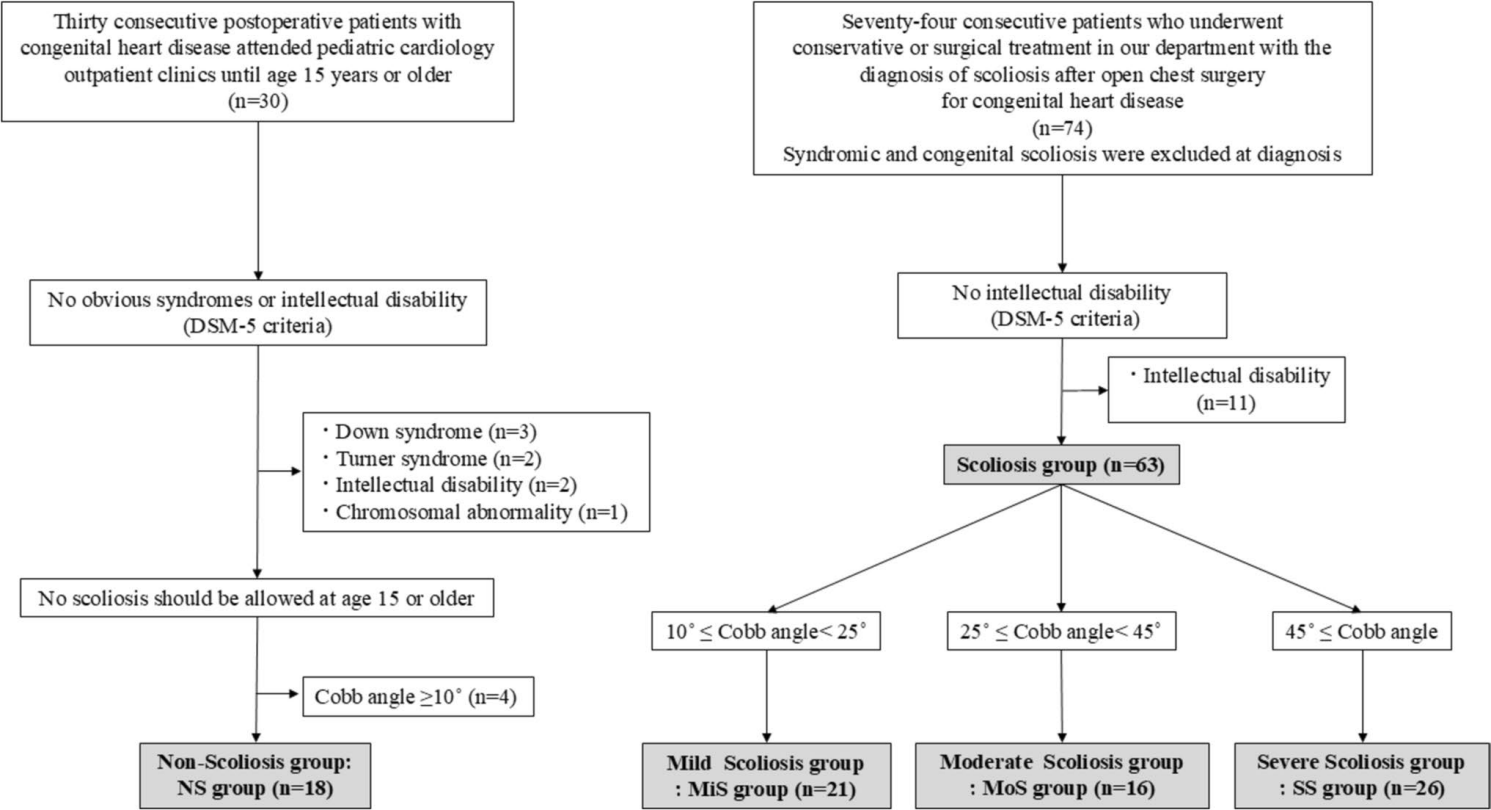
Study flowchart: patients who underwent open chest surgery for congenital heart disease and were followed until age 15 or older during the study period. The nonscoliosis group was used as a control, and the Scoliosis group included patients who were diagnosed with scoliosis after open heart surgery for congenital heart disease and who received conservative or surgical treatment. In the Scoliosis group, syndromic, congenital, and neuromuscular scoliosis were ruled out at diagnosis. Patients with obvious syndromes or intellectual disability, as evaluated by the Diagnostic and Statistical Manual of Mental Disorders, 5th Edition (DSM-5) criteria, were excluded. Furthermore, the scoliosis group was classified into three categories: the Mild Scoliosis group (10° ≤ Cobb angle < 25°), the Moderate Scoliosis group (25° ≤ Cobb angle < 45°), and the Severe Scoliosis group (Cobb angle ≥ 45°). The results were compared among the four groups

The cases of seventy-four consecutive patients who were diagnosed with scoliosis after open heart surgery for CHD and who underwent conservative or surgical treatment for scoliosis at two institutions until the age of 15 years between 2002 and 2021 were studied. This group was designated the Scoliosis group. Furthermore, we reviewed the cases of 30 consecutive postoperative patients with CHD who attended pediatric cardiology outpatient clinics at the authors’ institution until the age of 15 years or older from 1999 to 2021. These patients were categorized as the nonscoliosis group (NS group). In the NS group, patients with scoliosis with a Cobb angle of 10 degrees or greater on standing chest radiographs were excluded. CHD was diagnosed by both pediatric cardiologists and cardiovascular surgeons. Patients with obvious syndromes and intellectual disability according to the Diagnostic and Statistical Manual of Mental Disorders, 5th Edition (DSM-5) [12] were excluded. In the Scoliosis group, patients with syndromic, congenital, or neuromuscular scoliosis were excluded at diagnosis. Finally, 63 patients in the Scoliosis group and 18 patients in the NS group were included in the analysis. Furthermore, the Scoliosis group was classified into three groups as follows: Mild Scoliosis group (MiS group, 10° ≤ Cobb angle < 25°, n = 21), Moderate Scoliosis group (MoS group, 25° ≤ Cobb angle < 45°, n = 16) and Severe Scoliosis group (SS group, 45° ≤ Cobb angle, n = 26) (Fig. 1). Patient characteristics, such as sex, cardiac disease status, method of chest opening (sternotomy or thoracotomy), and number of chest openings, were collected by reviewing the patients’ electronic medical records. Cardiac disease was classified into five groups: right and left shunts, cyanotic lesions, obstructive lesions, single ventricles, and others. Patients in the Scoliosis group (Mis, Mos, and SS groups) were compared with those in the NS group. In the Scoliosis group, age at scoliosis diagnosis and scoliosis curvature were assessed.

Images were evaluated via the Cobb angle and cardiothoracic ratio (CTR) on the chest and whole-spine radiographs in the standing position. The Cobb angle was measured between the endplate of the upper vertebra and the endplate of the lower vertebra. Scoliosis was defined as a coronal curvature exceeding 10° and was graded as mild (10° ≤ Cobb angle < 25°), moderate (25° ≤ Cobb angle < 45°), or severe (45° ≤ Cobb angle). The CTR was estimated by dividing the cardiac diameter by the thoracic diameter.

Statistical analyses were conducted via JMP Pro statistical software (version 16.0; SAS Institute, NC, USA). Case analysis was performed, and the data are summarized as the mean ± standard deviation (SD) or the range for numeric variables. Fisher’s exact test was used to compare the sex distribution, type of scoliosis, and number of open chest surgeries between the NS and SS groups. The Wilcoxon rank-sum and Kruskal–Wallis tests were used to compare the CTR and ages at the final follow-up and the first CHD procedure between the NS and Scoliosis groups (Mis, MoS, and SS groups). A *p* < 0.05 indicated statistical significance.

## Results

### Comparison of patient background characteristics between the NS and Scoliosis groups

In the NS group, 18 patients (4 girls and 14 boys) met the inclusion criteria, whereas in the Scoliosis group, 63 patients (45 girls and 18 boys) met the inclusion criteria. The differences in patient characteristics between the NS and Scoliosis groups are shown in Table 1. In the NS group, there were 4 girls among the 18 children, whereas in the Scoliosis group, there were 45 girls among the 63 children, indicating a greater proportion of girls than in the NS group (*p* < 0.001). The mean CTR was 47.2 ± 4.9% in the NS group and 49.7 ± 6.6% in the Scoliosis group, but the difference was not significant. The age at the final follow-up in the NS group was 17.9 ± 2.3 years, and the age at surgery for scoliosis in the Scoliosis group was 19.6 ± 4.6 years. No significant differences were found between the two groups in the classification of cardiac disease (*p* = 0.07).

**Table 1:** Characteristics of the NS and Scoliosis groups

Parameter	NS group, n (n = 18)	Scoliosis group, n (n = 63)	*p* value
Sex			
Girls	4 (22.2%)	45 (71.4%)	<0.001
Age at final follow-up (years)	17.9 ± 2.3	19.6 ± 4.6	0.26
Cardiothoracic ratio (%)	47.2 ± 4.9	49.7 ± 6.6	0.16
Cardiac diagnosis			
Left to right shunt	10 (55.6%)	25 (39.7%)	0.07
Obstructive	0	6 (9.5%)	
Cyanotic	7 (38.9%)	16 (25.4%)	
Single ventricle lesion	0	15 (23.8%)	
Other	1 (5.6%)	1 (1.6%)	

*NS group* nonscoliosis group

Notes:

The age data at the final follow-up visit and the cardiothoracic ratio are presented as the means ± SDs

Fisher’s exact test was used to compare the sex distributions and cardiac diagnoses

The age data at the final follow-up and the cardiothoracic ratio were compared via the Wilcoxon rank sum test

Statistical significance was set at *p* < 0.05

### Characteristics of patients who underwent chest surgery in the NS and Scoliosis groups

The characteristics of the thoracic surgeries in the NS and Scoliosis groups are shown in Table 2. The mean age at the first CHD procedure was 3.5 ± 5.3 years in the NS group and 1.1 ± 1.8 years in the Scoliosis group, and the Scoliosis group was younger at the first surgery than the NS group (*p* = 0.04). With respect to the number of chest openings, more patients in the Scoliosis group required two or more chest openings (*p* < 0.01). In cases of median sternotomy, a full sternotomy was performed. For lateral thoracotomy, the approach was intercostal, and none of the ribs were resected. In this study, there were no cases of obvious rib deformity or fusion on X-ray images. With the open chest method, multiple sternotomies and combined approaches (at least one thoracotomy and one sternotomy) were more common in the Scoliosis group (*p* = 0.04). However, no significant between-group difference was found for thoracotomy or sternotomy alone, nor was there any difference between the groups who did and did not undergo thoracotomy. The side of the thoracotomy was not different.

**Table 2:** Chest surgeries in the NS and Scoliosis groups

Parameter	NS group, n (n = 18)	Scoliosis group, n (n = 63)	*p* value
Age at the first procedure for CHD (years)	3.5 ± 5.3	1.1 ± 1.8	0.04
Number of open chest surgeries			
≥2	4 (22.2%)	38 (60.3%)	<0.01
≥3	0	23 (36.5%)	<0.01
Method of chest opening			
One sternotomy only	13 (72.2%)	24 (38.1%)	0.04
One thoracotomy only	1 (5.6%)	2 (3.2%)	
Multiple sternotomies	4 (22.2%)	25 (39.7%)	
Combined approach (at least one thoracotomy and one sternotomy)	0	12 (19.0%)	
One sternotomy only/One thoracotomy only	13/1	24/2	1.0
Sternotomy only/At least one thoracotomy	17/1	49/14	0.17
Side of thoracotomy			
Left	1	6	0.54
Right	0	5	
Both	0	3	

*CHD* congenital heart disease, *NS group* nonscoliosis group

Notes:

The age at the first CHD procedure is presented as the mean ± SD

Age at the first procedure for CHD was compared with the Wilcoxon rank sum test

Data on the number of open chest surgeries and methods of chest opening were compared via Fisher’s exact test

Statistical significance was set at *p* < 0.05

### Comparison of patient background characteristics between the NS, MiS, MoS, and SS groups

The patients in the Scoliosis group (n = 63) were classified into three groups according to scoliosis severity: the MiS group (10° ≤ Cobb angle < 25°, n = 21), MoS group (25° ≤ Cobb angle < 45°, n = 16) and severe scoliosis group (45° ≤ Cobb angle, n = 26). The differences in patient characteristics among the 4 groups are shown in Table 3. With respect to sex, the proportion of girls in each group increased with increasing severity of scoliosis (*p* < 0.001). Age at the final follow-up also increased with increasing severity of scoliosis (*p* < 0.001), and each group was followed up until bone maturity. The CTR was not significantly different between the NS and Scoliosis groups (*p* = 0.16); however, among the four groups with varying scoliosis severity, the CTR significantly increased with increasing scoliosis severity (*p* = 0.038, NS group: 47.2 ± 4.9, MiS group: 47.4 ± 5.6, MoS group: 49.2 ± 4.4 and SS group: 51.8 ± 7.5). Regarding the treatment details, 20 patients in the MiS group (95.2%) and all patients in the MoS group underwent observational treatment, and in the SS group, 20 patients (95.2%) underwent surgery. There were significant differences among the four groups in the category of cardiac disease (left-to-right shunt, obstructive, cyanotic, single-ventricle lesion, and other) (*p* < 0.01), and a greater percentage of single-ventricle patients were observed to have increasing scoliosis severity.

**Table 3:** Characteristics of the NS, MiS, MoS and SS groups

Parameter	NS group, n (n = 18)	MiS group, n (n = 21)	MoS group, n (n = 16)	SS group, n (n = 26)	*p* value
Sex					
Girls	4 (22.2%)	11 (52.4%)	11 (68.8%)	23 (88.5%)	<0.001
Age at final follow-up (years)	17.9 ± 2.3	17.3 ± 2.2	18.3 ± 3.0	22.2 ± 5.4	<0.001
Type of treatment					
Observation	18(100%)	20 (95.2%)	16 (100%)	5 (19.2%)	
Bracing	–	1 (4.8%)	0	1 (3.8%)	
Surgery	–	0	0	20 (76.9%)	
Cardiothoracic ratio (%)	47.2 ± 4.9	47.4 ± 5.6	49.2 ± 4.4	51.8 ± 7.5	0.04
Cardiac diagnosis					
Left to right shunt	10 (55.6%)	12 (57.1%)	4 (25.0%)	9 (34.6%)	<0.01
Obstructive	0	4 (19.0%)	1 (6.3%)	1 (3.8%)	
Cyanotic	7 (38.9%)	5 (23.8%)	4 (25.0%)	7 (26.9%)	
Single ventricle lesion	0	0	6 (37.5%)	9 (34.6%)	
Other	1 (5.6%)	0	1 (6.3%)	0	

*NS group* nonscoliosis group, *MiS group* mild scoliosis group, *MoS group* moderate scoliosis group, *SS group* severe scoliosis group

Notes:

The age data at the final follow-up visit and the cardiothoracic ratio are presented as the means ± SDs

Fisher’s exact test was used to compare the sex distributions and cardiac diagnoses

The age data at the final follow-up and the cardiothoracic ratio were compared via the Kruskal–Wallis test

Statistical significance was set at *p* < 0.05

### Characteristics of patients who underwent chest surgery in the NS, MiS, MoS, and SS groups

The characteristics of the patients who underwent chest surgery in the NS, MiS, MoS, and SS groups are shown in Table 4. The mean age at first CHD surgery was 3.5 ± 5.3 years in the NS group, 1.3 ± 2.1 years in the MiS group, 1.0 ± 1.5 years in the MoS group, and 1.3 ± 1.6 years in the SS group, and no significant difference was found among the groups in the evaluation categorized by the degree of scoliosis progression (*p* = 0.26). Regarding the number of chest openings, a greater proportion of patients required more than two openings, particularly in the MoS and SS groups (*p* = 0.03). No significant differences were found among the four groups for thoracotomy or sternotomy alone, nor was there any difference between the groups with and without thoracotomy. Additionally, there was no difference in the side of the thoracotomy.

**Table 4:** Chest surgeries in the NS, MiS, MoS and SS groups

Parameter	NS group, n (n = 18)	MiS group, n (n = 21)	MoS group, n (n = 16)	SS group, n (n = 26)	*p* value
Age at the first procedure for CHD (years)	3.5 ± 5.3	1.3 ± 2.1	1.0 ± 1.5	1.0 ± 1.6	0.26
Number of open chest surgeries					
≥2	4 (22.2%)	11 (52.3%)	11 (68.8%)	16 (61.5%)	0.03
≥3	0	5 (23.8%)	8 (50.0%)	10 (38.5%)	<0.01
Method of chest opening					
One sternotomy only	13 (72.2%)	9 (42.9%)	5 (31.3%)	10 (38.5%)	0.14
One thoracotomy only	1 (5.6%)	2 (9.5%)	0	0	
Multiple sternotomies	4 (22.2%)	6 (28.6%)	8 (50.0%)	11 (42.3%)	
Combined approach (at least one thoracotomy and one sternotomy)	0	4 (19.0%)	3 (18.8%)	5 (19.2%)	
One sternotomy only/One thoracotomy only	13/1	9/2	5/0	10/0	0.38
Sternotomy only/At least one thoracotomy	17/1	15/6	13/3	21/5	0.33
Side of thoracotomy					
Left	1	4	0	2	0.54
Right	0	1	2	2	
Both	0	1	1	1	

*CHD* congenital heart disease, *NS group* nonscoliosis group, *MiS group* mild scoliosis group, *MoS group* moderate scoliosis group, *SS group* severe scoliosis group

Notes:

The age at the first CHD procedure is presented as the mean ± SD

Age at the first procedure for CHD was compared with the Kruskal–Wallis test

Data on the number of open chest surgeries and methods of chest opening were compared via Fisher’s exact test

Statistical significance was set at *p* < 0.05

### Spinal deformity in the scoliosis group

Table 5 shows the characteristics of the spinal deformities in the scoliosis groups (MiS, MoS, and SS groups). There was no significant difference in the age at scoliosis diagnosis among the three groups (*p* = 0.12). The curves were characterized by a single thoracic right convex curve, with the apical vertebrae around T8 or T9, with no significant differences among the three groups (type of scoliosis: *p* = 0.63, convexity: *p* = 0.45).

**Table 5:** Spinal deformity in the Scoliosis group

Parameter	Value, n			*p* value
Patients	MiS group, n (n = 21)	MoS group, n (n = 16)	SS group, n (n = 26)	
Cobb angle (degree)	17.0 ± 4.0	31.9 ± 5.3	70.7 ± 21.7	<0.001
Age at scoliosis diagnosis (years)	10.7 ± 3.7	9.0 ± 2.5	10.0 ± 3.9	0.12
Type of scoliosis				
Single thoracic	18 (85.7%)	12 (75.0%)	15 (57.7%)	0.63
Double thoracic	2 (9.5%)	3 (18.8%)	2 (7.7%)	
Double major	1 (4.8%)	1 (6.3%)	7 (26.9%)	
Thoraco-lumbar/Lumbar	0	0	2 (7.7%)	
Convexity in the main thoracic curve				
Right	17 (80.9%)	11 (68.8%)	23 (88.5%)	0.45
Apical vertebrae in the main thoracic curve				
T4	4 (19.0%)	2 (12.5%)	0	
T5	0	1 (6.3%)	1 (3.8%)	
T6	1 (4.8%)	0	1 (3.8%)	
T7	3 (14.3%)	2 (12.5%)	2 (7.7%)	
T8	3 (14.3%)	7 (43.8%)	10 (38.5%)	
T9	6 (28.6%)	3 (18.8%)	9 (34.6%)	
T10	2 (9.5%)	1 (6.3%)	0	
T11	1 (4.8%)	0	0	
T12	0	0	2 (7.7%)	
L1	1 (4.8%)	0	0	
L3	0	0	1 (3.8%)	

*MiS group* mild scoliosis group, *MoS group* moderate scoliosis group, *SS group* severe scoliosis group

Notes:

The Cobb angle and age at scoliosis diagnosis are expressed as the mean ± standard deviation

The Cobb angle and age at scoliosis diagnosis were compared with the Kruskal–Wallis test

The types of scoliosis and convexity in the main thoracic curve were compared via Fisher’s exact test

Statistical significance was set at *p* < 0.05

## Discussion

To our knowledge, this study is the first report of multiple open chest surgeries for severe CHD as factors leading to severe scoliosis, excluding obvious syndromes and intellectual disability. Although there have been previous reports comparing two groups with or without scoliosis [13], there have been no studies comparing controls without scoliosis over 15 years of age and patients with severe scoliosis. Sacco et al. [14] compared two groups of patients who underwent thoracotomy and who were aged 15 years or older with or without scoliosis (n = 28), but they did not compare the factors leading to severe scoliosis between the groups. In the present study, a comparison of the NS and Scoliosis groups, including the MiS, MoS, and SS groups, revealed that severe scoliosis was more frequently found in girls and patients who had undergone multiple open chest procedures. Patients who undergo multiple procedures do not have an increased risk of developing a spinal deformity [7]. Because of the small number of patients, multivariate analysis was not possible, and multiple chest openings could not be determined to be an independent factor. An examination of the 5 categories of cardiac diagnoses revealed that the incidence of a single ventricle significantly increased with increasing scoliosis severity. The high number of patients with single ventricles among the patients who underwent multiple surgeries may be another factor. Single ventricles and multiple open chest procedures have been reported to be associated with a slower progression of scoliosis [5], and the incidence of scoliosis has been reported to be associated with cardiomegaly and cyanotic conditions following Fontan completion surgery [15], although the underlying mechanism is not yet clear. Kaito et al. [4] reported that cardiomegaly was the sole predictor associated with scoliosis exceeding 45 degrees. Our results revealed no significant difference in the CTR between the NS and Scoliosis groups (*p* = 0.16). However, the CTR significantly increased with increasing severity of scoliosis among the four groups. These findings suggest that severe cardiac disease with cardiomegaly is a progressive factor. Cyanotic conditions could not be evaluated because the saturation of the room air at the last follow-up could not be confirmed for all patients. With respect to age at the time of the first CHD treatment, a comparison between the NS and Scoliosis groups revealed that the patients in the Scoliosis group were significantly younger. However, when the four groups classified by scoliosis severity were compared, no significant differences were observed. These findings suggest that early surgery for CHD may contribute to the development of scoliosis but not necessarily to its progression. In this study, scoliosis treatment was generally divided into follow-up and surgery, with orthotic treatment rarely used. While bracing is effective for adolescent idiopathic scoliosis [16], its efficacy for treating scoliosis following open chest surgery for CHD is unclear, as is the impact of bracing on cardiac function. Surgical treatment effectively corrects the deformity, but it carries risks such as infection and neurological complications [17]. Severe scoliosis in young patients can impair respiratory function [18] and lead to pain, reduced self-image, and diminished quality of life in adulthood [19]. Therefore, it is important to clarify the pathology and develop effective conservative treatments, such as bracing and early detection, as well as to develop safer and more effective surgical options. There are several reports of a constructive relationship between the thoracic spine and the sternum and thoracic cage [20, 21]. We focused on thoracic deformity as a cause of progression due to multiple open chest procedures and evaluated patients in the NS group (n = 5) and Scoliosis group (n = 17) in whom the thoracic deformity could be adequately confirmed by CT or MRI using the classification reported by Willital et al. [22]. However, no significant difference in thoracic morphology (pectus carinatum or excavatum) was found between the groups (data not shown). The relationship between thoracic deformities (pectus carinatum or excavatum) and scoliosis is still controversial [23–25], and direct and indirect stressful effects from multiple open chest procedures during early childhood may affect the morphogenesis and growth of the sternum and ribs [26]. In this study, not all patients were adequately evaluated for chest deformity via CT and MRI. Further research is needed to determine how multiple open chest procedures affect thoracic growth and spinal deformity. The curvature pattern in the Scoliosis group was similar to that in previous reports [3, 8, 10]. Other factors, including the timing of cardiac surgeries [3, 8], the use of sternotomy or thoracotomy [5, 8, 14, 27], and the type of surgical approach [11], have also been reported to be associated with scoliosis. The results of this study did not support these findings, and further studies, including a systematic review with a larger number of patients, are necessary.

It is important to acknowledge some limitations inherent in this study. First, this was a small, retrospective study with a limited population that focused on a rare disease other than adolescent idiopathic scoliosis. There is undeniable variation in the type of chest surgery and the severity of cardiac disease. To minimize the impact of these confounding factors, further investigations into the factors associated with the development and progression of scoliosis in this context are needed through larger prospective studies. Second, among patients who underwent multiple open chest surgeries, a factor that cannot be dismissed as a potential confounding influence was the greater number of girls. Although a small group, in boys only, the influence of multiple open chest surgeries was compared via Fisher’s exact test. The results showed that there were significantly more cases of multiple open chest surgery in the Scoliosis group (n = 18, multiple chest surgeries: n = 14) than in the NS group (n = 14, multiple chest surgeries: n = 3) (*p* < 0.01). This result was the same for the four groups (NS group: n = 14, 3 of whom were multiple open chest cases; MiS group: n = 10, 7 of whom were multiple open chest cases; MoS group: n = 5, all of whom were multiple open chest cases; and SS group: n = 3, 2 of whom were multiple open chest cases) classified by scoliosis severity (*p* < 0.01). Although the girl was identified as a predictive factor, the above results were also obtained in a sub-analysis of boys only, and therefore statistical evidence for that conclusion is not fully sound or supportive.

A better understanding of the relationship between multiple chest openings and factors leading to severe scoliosis, especially chest deformity, provides the key to elucidating the pathophysiology of scoliosis after open chest surgery for CHD.

## Conclusion

In conclusion, we investigated factors leading to postoperative scoliosis and progression to severe scoliosis in patients with CHD. Our results suggest that younger ages at first CHD surgery and undergoing multiple open chest surgeries may be factors in the development of advanced scoliosis. The evaluation of spinal deformity may be an important aspect of postoperative care for CHD patients, especially for pediatric patients who have undergone multiple open chest surgeries.

## Data Availability

The datasets analyzed during the current study are available from the corresponding author upon reasonable request.
